# Defective Toll-Like Receptors Driven B Cell Response in Hyper IgE Syndrome Patients With *STAT3* Mutations

**DOI:** 10.3389/fped.2021.738799

**Published:** 2021-11-05

**Authors:** Ruolan Gong, Jing Wu, Yingying Jin, Tongxin Chen

**Affiliations:** ^1^Division of Immunology, Institute of Pediatric Translational Medicine, Shanghai Children's Medical Center, School of Medicine, Shanghai Jiao Tong University, Shanghai, China; ^2^Allergy/Immunology Innovation Team, Shanghai Children's Medical Center, School of Medicine, Shanghai Jiao Tong University, Shanghai, China; ^3^Department of Rheumatology/Immunology, Shanghai Children's Medical Center, School of Medicine, Shanghai Jiao Tong University, Shanghai, China

**Keywords:** TLR7, TLR9, stat3, hyper IgE syndrome (HIES), B cell function

## Abstract

Autosomal dominant hyper-IgE syndrome (AD-HIES) is a rare inherited primary immunodeficient disease (PIDs), which is caused by *STAT3* gene mutations. Previous studies indicated a defective Toll-like receptor (TLR) 9-induced B cell response in AD-HIES patients, including proliferation, and IgG production. However, the other TLRs-mediated B cell responses in AD-HIES patients were not fully elucidated. In this study, we systematically studied the B cell response to TLRs signaling pathways in AD-HIES patients, including proliferation, activation, apoptosis, cytokine, and immunoglobulin production. Our results showed that the TLRs-induced B cell proliferation and activation was significantly impaired in AD-HIES patients. Besides, AD-HIES patients had defects in TLRs-induced B cell class switch, as well as IgG/IgM secretion and IL-10 production in B cells. Taken together, we first systematically reported the deficiency of TLRs driven B cell response in AD-HIES patients, which help to have a better understanding of the pathology of AD-HIES.

## Introduction

Hyper-IgE syndrome (HIES) is a kind of rare inherited primary immunodeficient disease (PIDs), which is characterized by elevated IgE levels, eczema, recurrent infections, and pneumonia. Both autosomal dominant (AD) and autosomal recessive (AR) modes of inheritance were reported in the patients with HIES, of which AD-HIES was the most common form. Loss-of-function (LOF) mutations of the gene encoding the signal transduction and activators of transcription 3 (STAT3) were identified as the cause of AD-HIES. In addition to the typical clinical manifestation of HIES mentioned above, AD-HIES patients were also reported to suffer from some non-immunological manifestations such as scoliosis, pathologic fractures, pneumatoceles, retained childhood dentition, coronary-artery aneurysms, brain lesions, craniofacial abnormalities (1, 2).

Signal transduction and activators of transcription 3, which belongs to the signal transducer and activator of transcription (STAT) family of signal responsive transcription factors, was reported to be involved in multiple biological functions, including cell proliferation, inflammation, differentiation, and survival. Therefore, it is not surprising that AD-HIES patients with *STAT3* mutations displayed a wide array of clinical features, which involves multiple organs in the body. According to researchers, STAT3 was involved in regulating T cells, B cells, neutrophils, and macrophages (3) in the immune system. For example, AD-HIES patients were reported to have reduced neutrophil chemotaxis and function, defective development and maintenance of T cell memory, reduced Th17 cells, reduced memory B cells, defective IL-10 and IL-21 signaling (3).

The innate immune system is the first defense by detecting and eliminating invading pathogens in humans. Pattern recognition receptors that widely exist in cells can further recognize pathogen-associated molecular patterns (PAMPs) such as liposomes, lipoproteins, proteins, and nucleic acids. Toll-like receptors (TLR), which belong to a special class of PAMPs, are involved in the recognition of molecular structures specific for microbial pathogens. Toll-like receptors are reported to express in antigen-presenting cells such as dendritic cells and macrophages, which play an important part in innate and adaptive immune responses (4). There are 10 types of TLRs reported in human beings, which can be grouped into two main categories: cell surface receptors that can recognize microbial membrane lipids including TLR1, 2, 4, 5, 6, 10, whereas receptors localized in the endosome including TLR3, 7, 8, 9 recognize microbial nucleic acids (4, 5). Among all the TLRs, TLR7 and TLR9 were demonstrated to be the widely expressed TLRs in human B cells, which were also regarded to be crucial to B cells' functions including proliferation, apoptosis, activation marker expression, and cytokine and immunoglobulin secretion (6–8). Studies showed the TLR9 agonists CpG oligodeoxynucleotides (CpG ODNs) could activate B cells response, promoting cell proliferation, plasma cells generation, cytokine secretion, and protecting them from apoptosis (6, 7, 9, 10). More than that, B cell activation mediated by TLR7 and TLR9 agonists can stimulate the production of IgG and IgM, making antibody shift to IgG2a and blocking the production of IgG1 and IgE (11–15). Of note, recent studies showed that STAT3 might play an important role in the B cell response mediated by TLRs, including cell proliferation, differentiation, and immunoglobulin production (16, 17). However, up to now, the other TLRs mediated B cell responses in AD-HIES patients were not fully elucidated. Therefore, more efforts are still needed to be done for further exploration.

Herein, we aimed to study the B cell responses upon TLRs agonists in AD-HIES patients, systematically evaluate the TLR-induced B cell response in patients with *STAT3* mutations, including proliferation, apoptosis, surface marker expression, memory B cell subsets, cytokine, and immunoglobulin secretion. Illustrating the role of STAT3 in TLRs-induced B cell responses in AD-HIES patients is the combination of basic and clinical medical studies and has great clinical value. It will not only help to give us a better understanding of the pathogenesis of AD-HIES but provide new ideas in target treatment development.

## Methods

### Patients and Control Samples

Six STAT3 mutant HIES patients (2 females and 4 males, age range 0.5–15 years; median 8.5 years) were enrolled in the experiment treated at Shanghai Jiaotong University-affiliated hospitals from June 2003 to August 2017. Patients met all of the following criteria: (1) Typical clinical symptoms of AD-HIES, including bacterial infections of the skin and lungs (especially pneumatocele and bronchiectasis), craniofacial abnormalities, mild traumatic fractures, scoliosis, retained childhood dentition, etc.; (2) Serum IgE levels > 2,000 IU/ml, and there were common allergen-specific IgE positives; (3) Patients who were determined to have a heterozygous dominant-negative mutation in the STAT3 gene by gene mutation analysis (Table 1). The detailed clinical features of these patients including history, immunophenotype characterization, and the treatment has already been published before by our group (1). At the same time, 12 healthy age-matched controls were recruited as the healthy controls, who had no obvious infection 4 weeks before blood donation, no blood products, and no immune modulators were used. All AD-HIES patients and healthy age-matched controls themselves or their guardians signed written informed consent and volunteered to be enrolled in the study. The study was approved by the local ethical institute (Shanghai Children's Medical Center, Shanghai Jiaotong University).

**Table 1:** Demographic, clinical, and laboratory characteristics of STAT3 mutant HIES patients reported in this study.

**ID^a^**	**Age at last evaluation (year)**	**Gender**	**Identified STAT3 mutation**	**Amino acid**	**Type**	**Immunological features**	**Non-immunological features**	**Lymphocytes subsets^b^**						**Immunoglobulin^b^**		
								**Lymphocytes** **(cells/μl)**	**CD3 cells** **(% of lymphocytes)**	**CD4 cells** **(% of lymphocytes)**	**CD8 cells** **(% of lymphocytes)**	**B cells** **(% of lymphocytes)**	**NK cells** **(% of lymphocytes)**	**Serum IgE (IU/ml)**	**Serum IgG (g/L)**	**Serum IgM (g/L)**
P1	5	Male	1144C>T	R382W	*De novo*	Pneumonia, pneumatocele, otitis media, sepsis, skin infections, pulmonary atelectasis, eczema	Distinctive facial features, retained primary teeth	4,400 (1,480–2,847)	70.7 (59.5–75.6)	40.2 (28.5–41.1)	18.4 (19.7–32.0)	26 (10.5–21.8)	3.2 (7.8–21.0)	6,710	9.2	1.1
P2	0.5	Male	1771A>G	K591E	*De novo*	Pneumonia, skin infections, lymph node abscesses, eczema	Distinctive facial features	5,490 (2,187–6,352)	84.2 (55.3–73.1)	48.9 (28.2–47.7)	35.4 (15.9–31.5)	4.5 (17.2–29.7)	5.7 (5.7–15.9)	2,510	7.7	0.7
P3	7	Female	1909G>A	V637M	*De novo*	Pneumonia, bronchiectasis, skin infections, thrush, EBV infection, eczema	Distinctive facial features, fracture	2,300 (1,424–2,664)	86.4 (60.1–74.1)	49.9 (26.2–40.8)	27.1 (19.7–34.1)	9.2 (10.2–20.1)	0.3 (9.0–22.2)	10,000	15.2	2.4
P4	15	Female	1144C>T	R382W	*De novo*	Pneumonia, bronchiectasis, skin infections, lymph node abscesses, pneumothorax, pleural thickening, eczema	Distinctive facial features, retained primary teeth, LVFT	3,790 (1,169–2,071)	77.6 (61.3–73.1)	35.2 (26.4–40.9)	33.6 (21.0–33.7)	8.1 (7.7–16.8)	4.5 (11.4–27.6)	2,500	11.6	2.9
P5	10	Male	1909G>A	V637M	*De novo*	Pneumonia, bronchiectasis, otitis media, skin infections, eczema	Distinctive facial features	2,200 (1,325–2,276)	74.4 (57.1–73.4)	42.2 (24.0–38.7)	29.9 (21.0–33.9)	20.1 (9.2–19.5)	4.4 (10.0–27.0)	26,000	12.3	1.8
P6	15	Male	1145G>A	R382Q	*De novo*	Pneumonia, bronchiectasis, skin infections, otitis media, lymph node abscesses, eczema	Distinctive facial features, retained primary teeth	2,700 (1,169–2,071)	87.8 (61.3–73.1)	43.6 (26.4–40.9)	40.3 (21.0–33.7)	8.7 (7.7–16.8)	2.3 (11.4–27.6)	22,900	14.1	0.9

a *P1, P3, P4, P5, and P6 in our study equal to P24, P7, P3, P10, and P15 in our formal study, respectively (1). Detailed information including history, clinical features, immunophenotype characterization, and the treatment can be found in that paper*.

b *Values at the time of investigation*.

*he number in round bracket shows the age specific reference value of healthy children in China*.

*The reference value of IgE, IgG, and IgM is <165 IU/ml, 7.0–16.0 g/L, and 0.4–2.3 g/L*.

### Cell Preparation

Human peripheral blood mononuclear cells (PBMCs) were isolated from 10 ml heparinized blood obtained from patients and healthy controls using Ficoll (Stemcell). Cells were cultured in RPMI-1640 containing 10% heat-inactivated fetal bovine serum (FBS) (Hyclone), 2 mM L-glutamine, 50 μg/ml streptomycin, and 100 U/ml penicillin (Hyclone).

### Proliferation Analysis

CFSE cell proliferation assay was used to monitor the B cell proliferation after treatment with various stimuli. Peripheral blood mononuclear cell were incubated in 5 μmol/l CFSE at 37°C for 10 min and washed with PBS supplemented with 5% FBS. CFSE-labeled PBMC (3 × 10^5^/well) were seeded in 96-well U bottom plates (BD) and divided into six groups: (1) control group; (2) TLR7/8 agonist (R848, 2.5 μg/ml, InvivoGen) group; (3) TLR9 agonist (CpG ODN 2006, called “CpG” here, 5 μg/ml, InvivoGen) group; (4) anti-IgM F(ab)_2_ fragments (anti-IgM, 0.5 μg/ml, Jackson Immuno Research) and sCD40L (1 μg/ml, R&D Systems) served as B cell activation stimulus; (5) R848 (2.5 μg/ml) combined with anti-human IgM (0.5 μg/ml) and sCD40L (1 μg/ml); (6) CpG (5 μg/ml) combined with anti-human IgM (0.5 μg/ml) and sCD40L (1 μg/ml). After 5 days, cells were harvested, washed, and stained with CD19-APC-H7 (BD) according to the manufacturer's instructions. Samples were then analyzed using BD FACS Canto II (BD, USA).

### Apoptosis Analysis

Human PMBC were plated in 96-well U bottom plates (3 × 10^5^/well) and divided into three groups: (1) control group; (2) R848 group (2.5 μg/ml); (3) CpG group (5 μg/ml). After 2 days, cells were harvested, washed, and stained with CD19-APCH7, Annexin V-APC, and 7AAD according to the manufacturer's instructions. Samples were then analyzed using BD FACS Canto II (BD, USA).

### Flow Cytometry

Human PMBC were plated in 96-well U bottom plates (3 × 10^5^/well) and divided into three groups: (1) control group; (2) R848 group (2.5 μg/ml); (3) CpG group (5 μg/ml). Cells were cultured for 3 days, then washed and stained with the following panels: B cell activation marker panel (CD19-FITC, CD80-PE, CD86-APC, CD40-APCH7, and HLA-DR-PE-Cy7); antibody panel (CD19-FITC, IgM-PE, and IgG-BV605); cytokine panel (CD19-FITC, IL-6-PE, and IL-10-APC); and isotype switch marker panel (CD19-FITC, CD27-PE-Cy7, and IgD-Percp-Cy5.5). For the intracellular staining panel, golgi plug (BD, USA) was added 5 h before harvesting the cells. After staining the surface marker, we used the Fixation/Permeabilization Kit (BD, USA) for intracellular marker staining. Flow cytometric analysis was performed using a Canto II flow cytometer (BD, USA). CS&T beads (BD, USA) were used in every experiment to maintain quality performance over time, Compensation was performed by using the negative control sample, single positive controls, and compensation beads (BD, USA) (for low expression markers). Flow cytometry data were analyzed using Flowjo 10.4 software (Treestar, USA).

### Statistical Methods

Graphpad Prism 6 software was used to generate graphs and process the experimental data. The data were compared by Student's *t*-test, one-way ANOVA, or Mann Whitney test. *P* < 0.05 indicated that the difference was statistically significant.

## Results

### Defective TLRs-Induced B Cells Proliferation in AD-HIES Patients With *STAT3* Mutations

**Figure 1:**
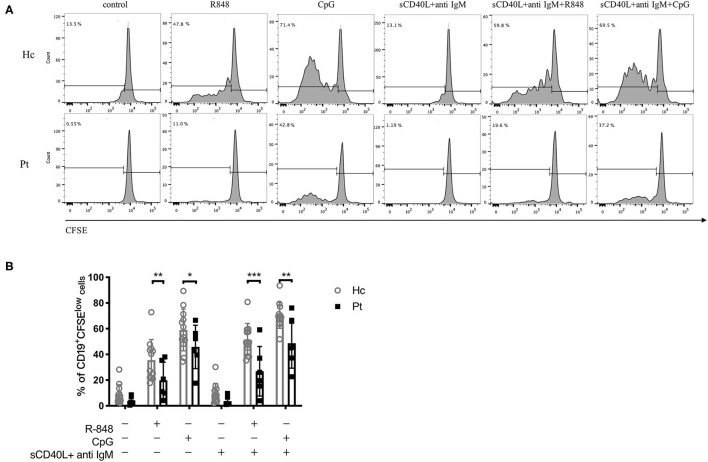
Defective R848 and CpG driven B cell proliferation in AD-HIES patients with *STAT3* mutations. PBMCs from AD-HIES patients and healthy controls were incubated with R848, CpG alone, or co-stimulation with sCD40L and F(ab′)2 anti-IgM for 5 days, then the B cell proliferation was determined by flow cytometry using CFSE. **(A)** A representative flow cytometric analysis of the B cell proliferation (results came from P6). **(B)** The results were expressed as the mean percentage of proliferative CD19^+^ B cell ± SD. Healthy controls (Hc, *n* = 12 each) and AD-HIES patients (Pt, *n* = 6 each). *P < 0.05, **P < 0.01, ***P < 0.001.

The proliferation of B cells is essential to adaptive immunity and is a key event to evaluate B cell function. To investigate the TLR-induced B cell proliferation in AD-HIES patients, CFSE labeled PBMCs from AD-HIES patients and age-matched healthy controls were stimulated with R848 or CpG (TLR7/8 or TLR9 agonists, respectively) alone, or in combination with F(ab') 2 fragments (anti-IgM) plus soluble CD40L (sCD40L). After 5 days' culture, CD19^+^ B cell proliferation rate was determined. As shown in Figure 1, significant proliferation of CD19^+^ B cell in TLRs stimulation group alone, as well as in combination with anti-IgM and sCD40L, were observed in both AD-HIE patients (*P* = 0.037 and *P* < 0.001 for R848 group and CpG group vs. control group, respectively; *P* = 0.003 and *P* < 0.001 for R848 and CpG together with anti-IgM plus sCD40L group vs anti-IgM plus sCD40L group, respectively), and healthy controls (all *P*-values were < 0.001). However, compared with healthy controls, AD-HIES patients had significantly decreased B cell proliferation rate upon TLRs stimulation alone, or in combination with anti-IgM and sCD40L. These data indicated that TLRs-induced B cell proliferation was defective in AD-HIES patients.

### Defective TLRs-Induced B Cell Activation in AD-HIES Patients

**Figure 2:**
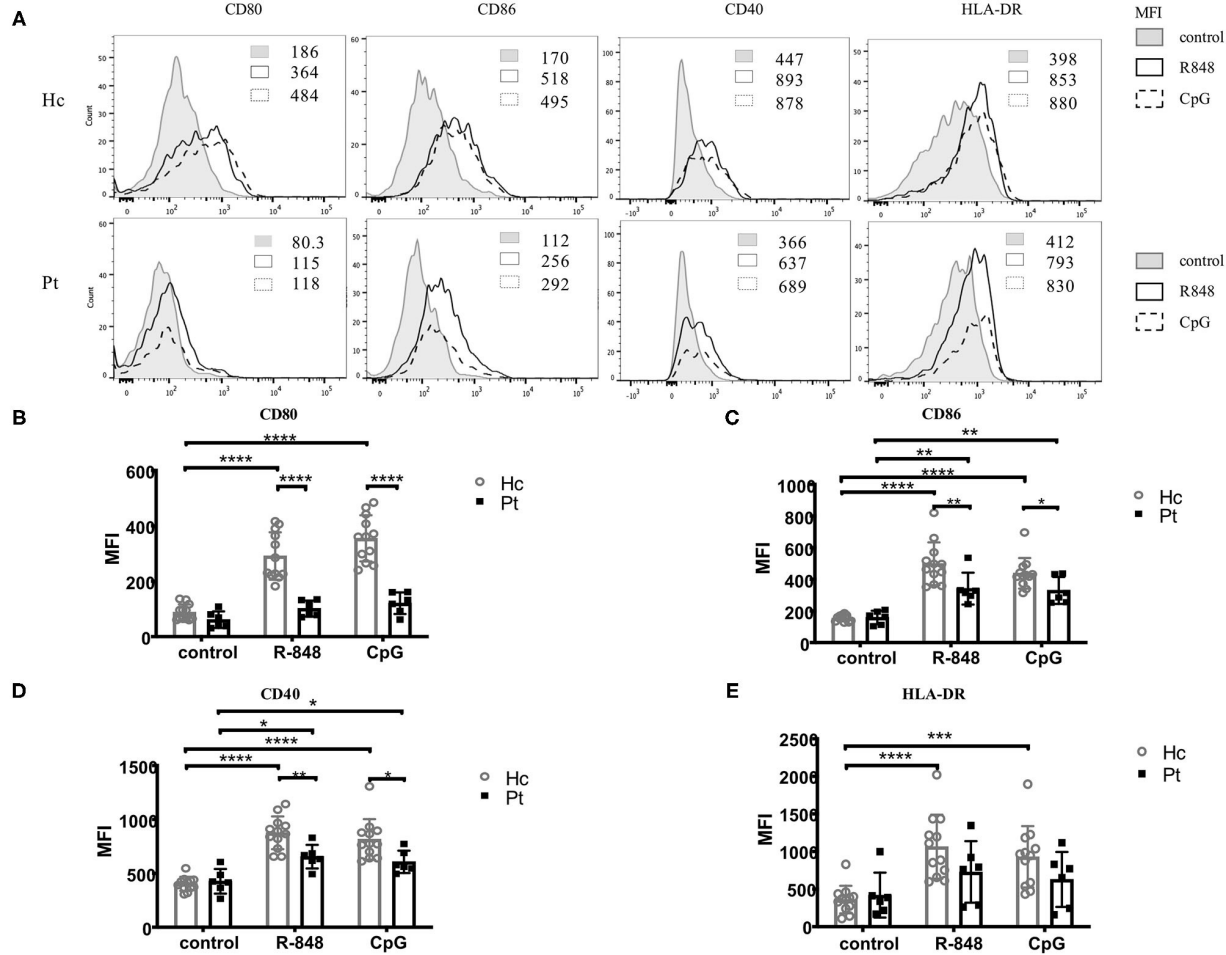
Defective R848 and CpG driven B cell activation in AD-HIES patients with *STAT3* mutations. PBMCs from AD-HIES patients and healthy controls were incubated with R848 or CpG for 3 days, then the expression of CD40, CD80, CD86, and HLA-DR molecules on the B cell surface was determined by flow cytometry. A representative flow cytometric analysis (P6) **(A)** and the mean fluorescence intensity (MFI) of CD80 **(B)**, CD86 **(C)**, CD40 **(D)**, and HLA-DR **(E)** were shown. Results were expressed as mean ± SD. Healthy controls (Hc, *n* = 12 each) and AD-HIES patients (Pt, *n* = 6 each) in this study. *P < 0.05, **P < 0.01, ***P < 0.001, ****P < 0.0001.

CD40, CD80 CD86, and MHCII are markers close related to B cell activation. Evaluation of the expression of these markers could help to assess B cell functions in the immune system. It was reported that B cell could be activated upon TLRs stimulation, with an upregulation of CD40 molecules, as well as some co-stimulation molecules including CD80, CD86, and HLA-DR on B cell surface (18, 19). As reported before, the expression of CD40, CD80, CD86, and HLA-DR on B cell surface were all significantly upregulated upon TLRs stimulation in healthy controls. However, only the expression of CD86 and CD40 on B cell surface was upregulated upon TLRs stimulation in AD-HIES patients; while the expression of CD80 and HLA-DR was not affected (Figure 2). Moreover, compared with healthy controls, TLRs-induced up-regulation of CD40, CD80, and CD86, but not MHCII was significantly decreased on B cell surface in AD-HIES patients, which suggested a defective TLRs-induced B cell activation in AD-HIES patients.

### Defective TLR-Induced Intracellular IgG, IgM, and IL-10 Secretion in B Cells From AD-HIES Patients

**Figure 3:**
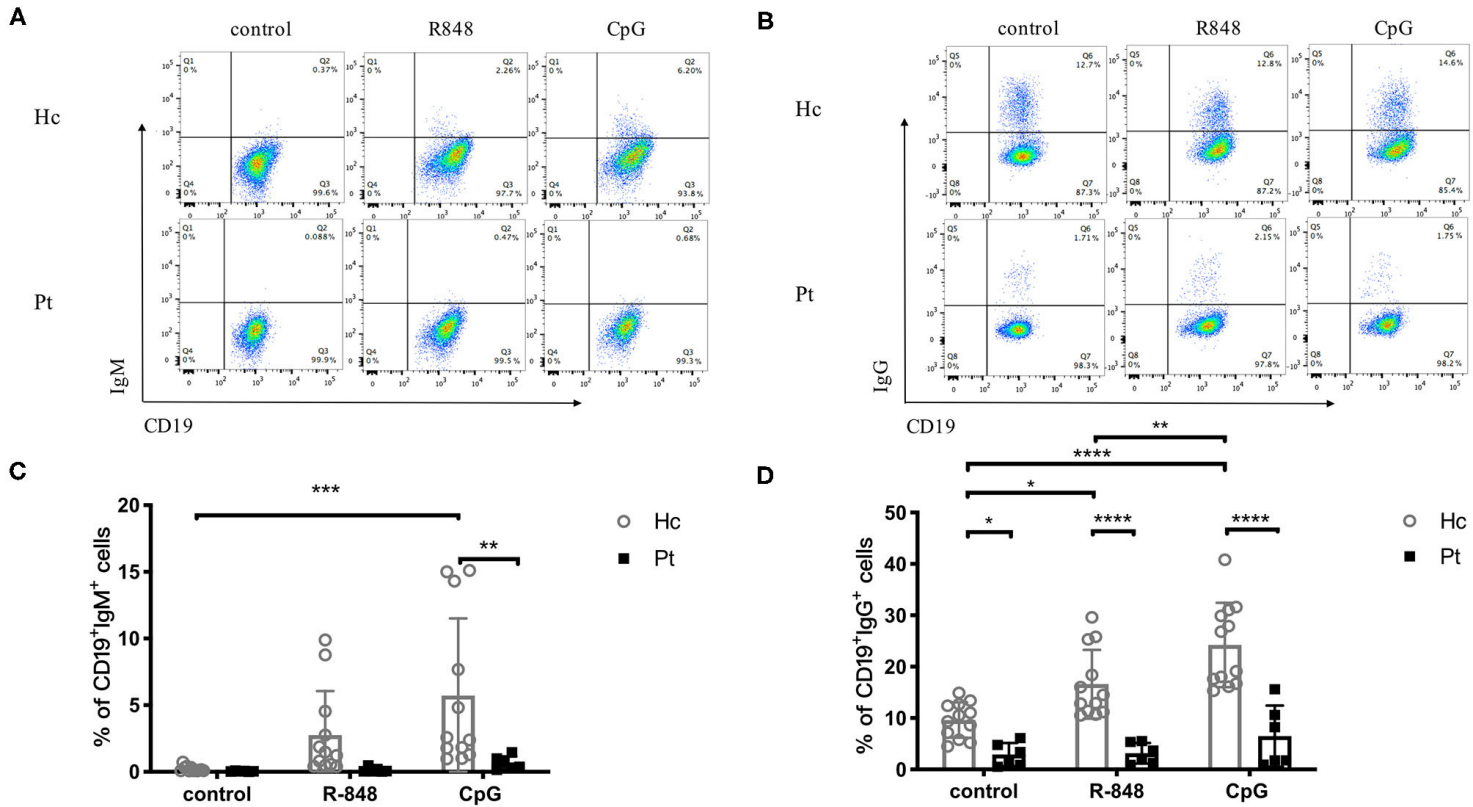
Reduced IgM and IgG production induced by R848 and CpG in AD-HIES patients with *STAT3* mutations. PBMCs from AD-HIES patients and healthy controls were incubated with R848 or CpG for 3 days, then the productions of IgM and IgG in B cells were evaluated by intracellular flow cytometry. Representative intracellular flow cytometry of IgM **(A)** and IgG **(B)** production in B cell after R848 or CpG stimulation (results came from P6). The results are expressed as the mean percentage of IgM positive **(C)** and IgG positive **(D)** in CD19^+^ B cell ± SD. Healthy controls (Hc, *n* = 12 each) and AD-HIES patients (Pt, *n* = 6 each) in this study. *P < 0.05, **P < 0.01, ***P < 0.001, ****P < 0.0001.

Antibodies secretion is the most important part of the adaptive immune system and a signal of B cell differentiation and activation. Recently, a defective TLR9-induced IgG secretion by PBMC was reported in AD-HIES patients (16). In the present study, we further detected TLRs-induced IgG, as well as IgM secretion in B cells from AD-HIES patients. Our results showed that, compared with healthy controls, AD-HIES patients had significantly decreased TLRs-induced IgM and IgG secretion (Figure 3).

**Figure 4:**
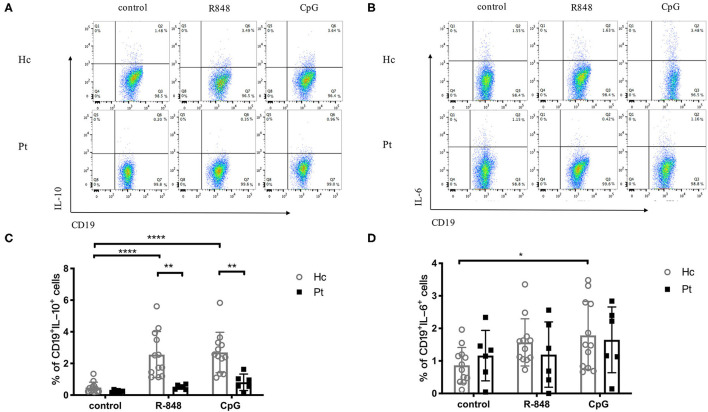
Decreased IL-10 production in B cells induced by R848 and CpG in AD-HIES patients with *STAT3* mutations. PBMCs from AD-HIES patients and healthy controls were incubated with R848 or CpG for 3 days, then the production of IL-10 in B cells was evaluated by intracellular flow cytometry. Representative flow cytometry of IL-10 **(A)** and IL-6 **(B)** in B cell after R848 or CpG stimulation (results came from P6). The results are expressed as the mean percentage of IL-10 positive **(C)** and IL-6 positive **(D)** in CD19^+^ B cell ± SD. Healthy controls (Hc, *n* = 12 each) and AD-HIES patients (Pt, *n* = 6 each) in this study. *P < 0.05, **P < 0.01, ***P < 0.001, ****P < 0.0001.

B cells are capable of producing cytokines, which depends on their differentiation state and activation conditions. The TLR-induced IL-6 and IL-10 secretion in B cells from AD-HIES patients was determined in this study. As shown in Figure 4, R848 and CpG could significantly increase intracellular IL-10 secretion in B cells from healthy controls (Figures 4A,C), but they had little effects on the IL-6 secretion (Figures 4B,D). Of note, compared with age-matched healthy controls, a defective TLRs-induced IL-10 secretion in B cells was observed in AD-HIES patients. These results indicated that the STAT3 might be involved in TLR-induced IgG and IgM secretion, as well as IL-10 secretion in B cells.

### Defective TLR-Induced B Cell Class Switch in AD-HIES Patients

**Figure 5:**
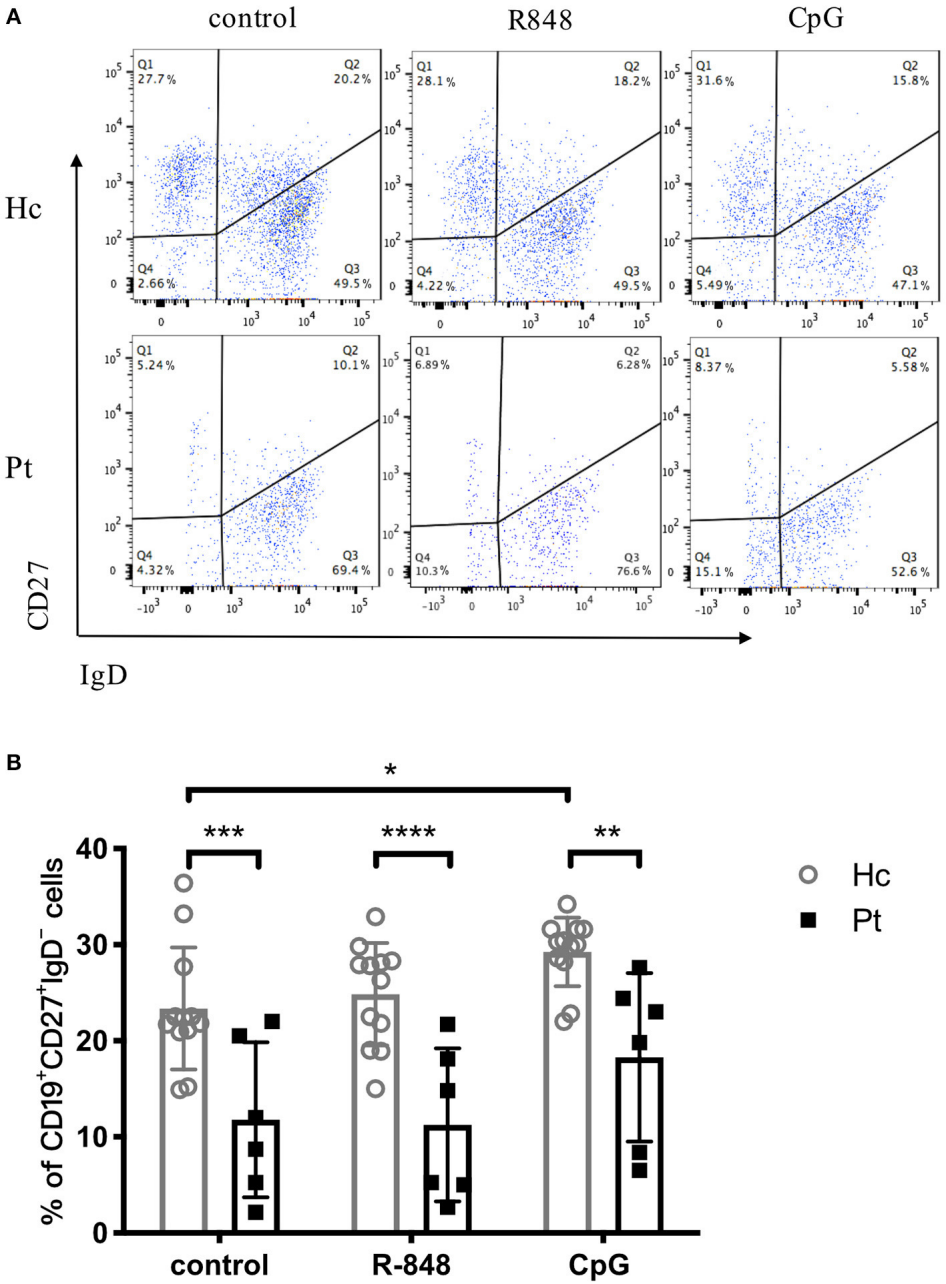
Reduced number of switched memory B cells in AD-HIES patients with *STAT3* mutations. PBMCs from AD-HIES patients and healthy controls were incubated with R848 or CpG for 3 days, then the expression of CD27 and IgD on the B cell surface was determined by flow cytometry. Representative flow cytometric results **(A)** and transformed memory CD27^+^IgD^−^ cells **(B)** in B cell after R848 or CpG stimulation (results came from P6). The results are expressed as the mean percentage of CD27^+^IgD^−^ cells in CD19^+^ B cell ± SD. Healthy controls (Hc, *n* = 12 each) and AD-HIES patients (Pt, *n* = 6 each) in this study. *P < 0.05, **P < 0.01, ***P < 0.001, ****P < 0.0001.

Immunoglobulin isotype switch occurs after activation of B cells, which is a crucial part of functional antibody secretion followed by B cell proliferation. Autosomal dominant hyper-IgE syndrome patients were previously reported to have significantly decreased CD27^+^ memory B cells, including both CD27^+^IgD^−^ class-switched memory B cells and CD27^+^IgD^+^ non-class-switched memory B cells (20). The B-cell subset distribution observed in the present study was consistent with the previous reports (Figure 5). The results showed that CpG stimulation significantly increased the percentage of CD27^+^IgD^−^ class-switched memory B cells in healthy controls. However, there was no significantly increased CD27^+^IgD^−^ class-switched memory B cells observed in AD-HIES patients after CpG stimulation, which indicated that STAT3 might be involved in B cell antibody isotype conversion (Figure 5). However, R848 stimulation, which had a small effect on the B cell isotype switch, could be seen as a negative control (Figure 5).

### TLRs-Induced B Cell Apoptosis Was Not Affected in AD-HIES Patients

**Figure 6:**
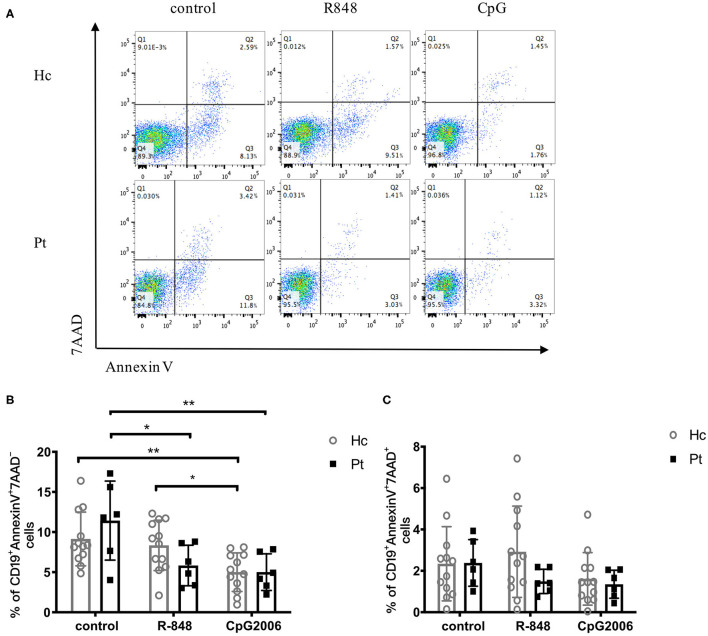
Similar apoptosis of B cell induced by R848 and CpG in AD-HIES patients and healthy controls. PBMCs from AD-HIES patients and healthy controls were incubated with R848 or CpG for 2 days, then the apoptosis of B cell was evaluated by flow cytometry. **(A)** Representative flow cytometry of B-cell apoptosis after d R848 or CpG stimulation (results came from P6). The percentages of B cell apoptosis **(B)** and necrosis **(C)** in healthy controls (Hc, *n* = 12 each) and AD-HIES patients (Pt, *n* = 6 each) were shown as the mean ± SD. Apoptotic B cells: AnnexinV^+^7AAD^−^; Necrotic B cells: AnnexinV^+^7AAD^+^. *P < 0.05, **P < 0.01.

Induction of B cell apoptosis and its regulation are likely to play important roles in humoral immunity. Apoptosis and necrosis of B cells after TLR stimulation were analyzed in AD-HIES patients and healthy controls in this study. As shown in Figure 6, R848 and CpG decreased the apoptosis of B cells in both AD-HIES patients and age-matched healthy controls. However, there was no significant B cells' apoptosis and necrosis difference between the two groups, which indicated that B cell apoptosis was not affect in AD-HIES patients (Figure 6).

## Discussion

Autosomal dominant hyper-IgE syndrome with STAT3 deficiency is an extremely rare primary immunodeficiency disease with a prevalence of nearly 0.64–1/1,000,000 (1). One of the most striking clinical features for patients with LOF *STAT*3 mutations is recurrent infections. Therefore, it would be easy to deduce that the STAT3 signaling pathway played a vital role in human immune systems. Remarkably, the decreased memory B cells reported in AD-HIES patients throw light on the role of STAT3 in B cell development and function. Previous research has shown that STAT3 was downstream of DOCK8 in TLR9-mediated B cell proliferation and IgG secretion. However, the TLRs-induced B cell response in AD-HIES patients has not been fully explored yet. In the present study, we demonstrated STAT3 participated in TLR7/9-induced B cell response, including proliferation, activation, IgM/IgG secretion, IL-10 secretion, and B cell class switch.

Anti-human IgM antibodies can bind to IgM receptors expressed on the surface of B cell membranes and activate cross-linking of surface receptors, signal transduction, and antigen presentation. It has been reported that sCD40L acts similarly to CD40L and can help activate B cells (21). CD40 and CD40L signal pathways provide the second signal for B cell activation, which is crucial for the growth, differentiation, and proliferation of B cells (21). A wide variety of studies have shown that sCD40L and anti-human IgM promoted B cell activation induced by R848 and CpG (22–24). It is reported that patients with *DOCK8* mutations had B cell proliferation defects induced by CpG, in which STAT3 was downstream of DOCK8 (16). In this study, we further demonstrated that, in addition to CpG, R848 could also induce B cell proliferation both in healthy controls and AD-HIES patients. However, compared with the healthy controls, the TLRs-induced B cell proliferation was significantly defective in AD-HIES patients. These results indicated that STAT3 participated in TLRs-induced B cell proliferation. Apart from the STAT3 signaling pathway, other pathways were also reported to be involved in the TLRs-induced B cell proliferation. For example, Fruman et al. reported that subunit p85α of phosphoinositide 3-kinase (PI3K) was an important enzyme for B cell differentiation and proliferation (25). Defects in B-cell proliferation in the TLR-MyD88-STAT3 signal in AD-HIES patients might be related to p85α in PI3K, which is worthwhile to further explore.

It is known from the previous study that CD80 and CD40 expression on the surface of naive B cells from healthy controls can be significantly up-regulated even after stimulation with CpG (6). In this study, we showed that the B cell surface CD80, CD86, and CD40 expression in AD-HIES patients induced by R848 and CpG was significantly decreased than that in healthy controls, which implied the connection between STAT3 and TLRs-induced B cell activation. Moreover, given the well-demonstrated role of CD80 and CD86 in T-B cells' cooperation, we can speculate that the TLR-STAT3 signaling pathway might also be involved in the interaction of B cells and T cells. In AD-HIES patients, Th17 differentiation disorder results in a defect of IL-17 secretion and neutrophil proliferation and chemotaxis abnormal, which makes patients vulnerable to Candida infection (26, 27). The impaired CD80 and CD86 expression on B cells in AD-HIES patients might be related to their impaired CD4^+^ T cell differentiation. CD40 can bind to CD40L which express on T cells, providing a second signal for B cell activation. CD40 expression on the surface of B cells reflects the degree of B cell activation. *STAT3* mutation may affect B cells' second signal transduction by down-regulating CD40 expression. HLA-DR expression in healthy controls was significantly up-regulated only after stimulation, but not in patients. Previous studies had a similar result that HLA-DR expression in naive B cells was significantly increased induced by CpG in healthy controls (6). Whereas, TLR-STAT3 signaling pathway had no close correlation to B cell antigen presentation.

IgM and IgG secretion in healthy human B cells increased after being stimulated with R848 and CpG. However, AD-HIES patients had no significant changes in IgM and IgG secretion stimulated with R848 and CpG. Similar results were obtained in the studies of Wei Jiang and Mark Glaum, which showed that R848 and CpG can stimulate IgM and IgG secretion in naive B cells in healthy controls (6, 8). These results proved that the functional deficiency of antibody secretion in the patient has no connection to the large population of naive B cells in patients. Therefore, the TLR-STAT3 signal pathway might be related to the secretion of IgM and IgG. Moreover, Giardino reported that patients with NF-κB deficiency can not secrete IgG antibodies after CpG stimulation, which proved that the TLR9-NF-κB signal pathway is also involved in B cell antibody secretion (28). Previous studies showed that the total serum IgM and IgG concentrations in AD-HIES patients were similar to normal people (1). However, the detection method in this study is different from most studies. The lower secretion of IgM and IgG in patients during the study might be due to the abnormal transmission of the TLR9-STAT3 signal pathway, which weakened the immune response, slowed the secretion of antibodies, shifted the peak of antibody secretion, then led to patients' repeated Candida infection.

IL-6 secretion increased slightly in healthy controls after CpG in our study, which is associated with NF-κB and STAT3 phosphorylation. The expression of STAT3 protein and STAT3 phosphorylation in IL-6 knockdown mice was significantly reduced (29). At the same time, other studies had found that IL-6 secretion was reduced in samples from AD-HIES patients, and *STAT3* deficiency led to impaired IL-6 signal pathway (30). The *STAT3* mutation had no significant effect on IL-6 secretion in B cells but inhibited serum total IL-6 secretion. The reason might be the fact that the production of IL-6 in the patient's serum was mainly produced by T cells. Hence, patients had no significant change in B cells' IL-6 secretion. The amount of IL-10 secreted by healthy controls' B cells was very low. IL-10 secretion increased significantly induced by R848 and CpG in the control group, but not in patients. Therefore, the TLR-MyD88-STAT3 signal pathway might be involved in IL-10 secretion by B cells. IL-10 was mainly secreted by Th2 cells and other immune cells including B cells, dendritic cells, and NK cells (31). *STAT3* deficiency caused up-regulation of Th1 cytokines and down-regulation of the anti-inflammatory factor IL-10 (32). IL-10 secretion by dendritic cells and Th17 cells can also be regulated by the TLR9-MyD88-ERK-STAT3 signal pathway, which was consistent with the results of our study.

CD27 is expressed on the surface of memory B cells. Two types of BCRs are expressed on the surface of mature primary B cells, which are mIgM and mIgD. The mIgD of activated B cells or memory B cells gradually disappeared. After being stimulated by CpG, CD27^+^IgD^−^B cells were significantly increased in healthy controls. The R848 stimulation did not induce any class-switching in healthy controls, which made the outcome of this experiment not that informative. However, we observed that CpG stimulation did induce class-switching in healthy controls, which suggested that the TLR9 pathway might contribute to B cell isotype switch in healthy controls. On the contrary, stimulation with the TLR9 agonist did not trigger B class-switching in HIES patients with STAT3 mutations, indicating that inhibition of STAT3 might be involved in TLR9-induced B cell isotype switch. Therefore, R848 stimulation, which had a small effect on the B cell isotype switch, could be seen as a negative control. Studies showed that CpG can lead to B cells antibody isotype switching through innate immune pathways (13, 33), or the up-regulation of MyD88 expression (13). Further studies about TLR9-induced B cell isotype switching remains to be done. Early studies showed that the number of memory B cells in AD-HIES patients was significantly lower than that in normal people (1, 20), which was also confirmed by the significantly lower number of switched memory B cells in patients in our study.

After CpG stimulation, healthy human B cell apoptosis was significantly reduced, while R848 stimulation was not. In AD-HIES patients, both R848 and CpG significantly reduced B-cell apoptosis. A study by Wei Jiang also reported that R848 and IL-4 significantly reduced B cell apoptosis. However, the *STAT3* mutation did not affect B cell apoptosis which suggested that there might be other TLR signaling pathways that participate in saving B cell apoptosis of patients with *STAT3* mutations (6).

Despite having mutations in different domains of STAT3, all patients showed similar kinds of defective B cell function *ex-vivo* results. It was reported that these mutations were all LOF *STAT3* mutations and led to impaired STAT3 signaling (34, 35), which might have a similar effect on patients' B cell function.

In conclusion, we found that the B cell proliferation, CD80, CD86, and CD40 expression, IgG and IL-10 secretion, and switched memory B cell subsets were defective in AD-HIES patients induced by TLR7 and/or TLR9 agonist. We studied and found the connection between the TLR-STAT3 signal pathway and B cell function from a clinical perspective by using AD-HIES patients' cells. Therefore, abnormal TLR-MyD88-STAT3 signal pathway might participate in the pathogenesis of B cell dysfunction and a series of immune phenotypes in AD-HIES patients. Whereas, the detailed pathogenesis of *STAT3* mutation in the TLR-MyD88-STAT3 signal pathway needs to be further studied. At the same time, this signal pathway might also be related to the pathogenesis of other primary immunodeficiency diseases, such as hyper IgM syndrome, and chronic granulomatosis. Further research about the TLR-MyD88-STAT3 signal pathway will help us to reveal every small link of AD-HIES immune deficiency, and develop targeted treatments to reduce the pain for children currently suffering from the disease.

## Data Availability Statement

The original contributions presented in the study are included in the article/supplementary material, further inquiries can be directed to the corresponding author/s.

## Ethics Statement

The studies involving human participants were reviewed and approved by the Local Ethical Institute (Shanghai Children's Medical Center, Shanghai Jiaotong University). Written informed consent to participate in this study was provided by the participants' legal guardian/next of kin.

## Author Contributions

RG, JW, YJ, and TC contributed to the design and implementation of the research, patients' organization, analysis of the results, and the writing of the manuscript. All authors contributed to the article and approved the submitted version.

## Funding

This research was supported by grants from the National Natural Science Foundation of China (81871303, 81701626, 81571605, and 81273314).

## Conflict of Interest

The authors declare that the research was conducted in the absence of any commercial or financial relationships that could be construed as a potential conflict of interest.

## Publisher's Note

All claims expressed in this article are solely those of the authors and do not necessarily represent those of their affiliated organizations, or those of the publisher, the editors and the reviewers. Any product that may be evaluated in this article, or claim that may be made by its manufacturer, is not guaranteed or endorsed by the publisher.
